# Wnt16 Signaling Is Required for IL-1β-Induced Matrix Metalloproteinase-13-Regulated Proliferation of Human Stem Cell-Derived Osteoblastic Cells

**DOI:** 10.3390/ijms17020221

**Published:** 2016-02-06

**Authors:** Nobuaki Ozeki, Makio Mogi, Naoko Hase, Taiki Hiyama, Hideyuki Yamaguchi, Rie Kawai, Ayami Kondo, Kazuhiko Nakata

**Affiliations:** 1 Department of Endodontics, School of Dentistry, Aichi Gakuin University, Nagoya, Aichi 464-8651, Japan; hanako@dpc.agu.ac.jp (N.H.); meijichelsea81@gmail.com; (T.H.); ag123d27@dpc.agu.ac.jp (H.Y.); riechan@dpc.agu.ac.jp (R.K.); kazukun@dpc.agu.ac.jp (K.N.); 2 Department of Medicinal Biochemistry, School of Pharmacy, Aichi Gakuin University, Nagoya, Aichi 464-8650, Japan; makio@dpc.agu.ac.jp (M.M.); ayami@dpc.agu.ac.jp (A.K.)

**Keywords:** Wnt16, matrix metalloproteinase, osteoblast, interleukin-1β, cell proliferation

## Abstract

We established a differentiation method for homogeneous α7 integrin-positive human skeletal muscle stem cell (α7^+^hSMSC)-derived osteoblast-like (α7^+^hSMSC-OB) cells, and found that interleukin (IL)-1β induces matrix metalloproteinase (MMP)-13-regulated proliferation of these cells. These data suggest that MMP-13 plays a potentially unique physiological role in the regeneration of osteoblast-like cells. Here, we examined whether up-regulation of MMP-13 activity by IL-1β was mediated by Wingless/int1 (Wnt) signaling and increased the proliferation of osteoblast-like cells. IL-1β increased the mRNA and protein levels of Wnt16 and the Wnt receptor Lrp5/Fzd2. Exogenous Wnt16 was found to increase MMP-13 mRNA, protein and activity, and interestingly, the proliferation rate of these cells. Treatment with small interfering RNAs against Wnt16 and Lrp5 suppressed the IL-1β-induced increase in cell proliferation. We revealed that a unique signaling cascade IL-1β→Wnt16→Lrp5→MMP-13, was intimately involved in the proliferation of osteoblast-like cells, and suggest that IL-1β-induced MMP-13 expression and changes in cell proliferation are regulated by Wnt16.

## 1. Introduction

Matrix metalloproteinases (MMPs) play central roles in cell proliferation, migration, differentiation, angiogenesis, apoptosis, and host defenses. Deregulation of MMPs has been implicated in many diseases including rheumatoid arthritis (RA), chronic ulcers, encephalomyelitis, and cancer [1,2,3]. Human collagenase-3 is an enzyme encoded by the *MMP13* gene. During embryonic development MMP-13 is expressed in the skeleton to restructure the collagen matrix for bone mineralization. MMP-13 is highly overexpressed in pathological situations such as carcinomas RA, and osteoarthritis (OA). Furthermore, MMP-13 may be involved in the articular cartilage turnover and cartilage pathophysiology associated with OA. Dramatic up-regulation of MMP-13 by inflammatory cytokines, such as interleukin (IL)-1β, has been observed in chondrocytes [4]. We previously reported that MMP-13 accelerates bone remodeling following the development of periradicular lesions [5], and presented evidence suggesting that MMP-13 plays a potentially unique physiological role in wound healing and the regeneration of alveolar bone. Because alveolar bone tissue consists predominantly of osteoblasts, these cells may represent a potential target cell type for new therapeutic strategies to mitigate these disease states. Moreover, we have reported that the proinflammatory cytokine IL-1β induces MMP-13 activity in purified osteoblast-like cells derived from human stem cells [6]. IL-1 plays an important role in proliferation of cells at sites of tissue injury at relatively low concentrations [1]. However, the signaling cascade underpinning such stimulation is uncharacterized.

Wingless/int1 (Wnt) signaling plays an important role in the development and maintenance of many organs and tissues by regulation of cell growth, differentiation, functions, and death via various signaling pathways [7]. Noncanonical Wnt signaling, which is independent of β-catenin, may also play a role in bone formation through promotion of osteoblastic differentiation [3]. Wnt signaling might be involved in the destruction of temporomandibular joint condylar cartilage after experimentally induced OA [8]. Several Wnt isoforms (Wnt5a, Wnt7a, and Wnt11) are involved in IL-1-induced differentiation of articular chondrocytes [9,10]. In addition, Wnt16 is a key factor in developing long bones, is a member of the wingless-type MMTV integration site family, which mediates signaling via canonical or non-canonical Wnt pathways. Interestingly, several independent human genome-wide association studies have identified the Wnt16 locus as an important genetic determinant contributing to variations in the bone mineral density of humans [11,12,13]. For example, Zheng *et al.* [14] found significant associations between single nucleotide polymorphisms in the Wnt16 locus and cortical thickness in a genome-wide association study of five cohorts including 5672 participants. These findings indicate that Wnt16 is crucial for the development of bones and regulation of bone mass. However, the exact role of Wnt16 in human osteoblast development remains unclear. Furthermore, although Wnt5a has been linked to the regulation of MMP-1, MMP-3, and MMP-7 in various cell types [15], there is currently no evidence indicating that Wnt16 influences the expression of MMP-13 in human osteoblasts. We previously demonstrated that IL-1β-induced MMP-3-regulated proliferation of mouse odontoblastic cells is mediated by the Wnt5 signaling pathway [16].

Human osteoblastic cells are commercially available as osteosarcoma-derived cell lines including MG-63, Hos, U2Os, and SaoS-2. Because of the challenges associated with obtaining sufficient numbers of purified human osteoblasts, studies have yet to focus on purified osteoblasts treated with IL-1β as a model of early-phase inflammation. Therefore, we employed purified osteoblast-like cells derived from α7 integrin-positive human skeletal muscle stem cells (α7^+^hSMSCs) [17] as an appropriate cell model to examine the mechanism of wound healing and cell survival *in vitro*. These cells are an excellent *in vitro* model to examine the mechanisms of wound healing.

Here, we examined whether Wnt signaling is associated with the expression of MMPs during osteoblast activity, which may occur in inflamed bone fragility fractures. Our study of human skeletal muscle stem cell-derived osteoblast-like cells aimed to delineate the degree of involvement of Wnt16 in the expression of MMPs and the molecules that regulate this process. We show, for the first time, that Wnt16 up-regulates MMP-13 in osteoblast-like cells, which induces cell proliferation.

## 2. Results

### 2.1. IL-1β Induction of Wnt16 mRNA and Protein Expression in α7^+^hSMSC-OB Cells

**Figure 1:**
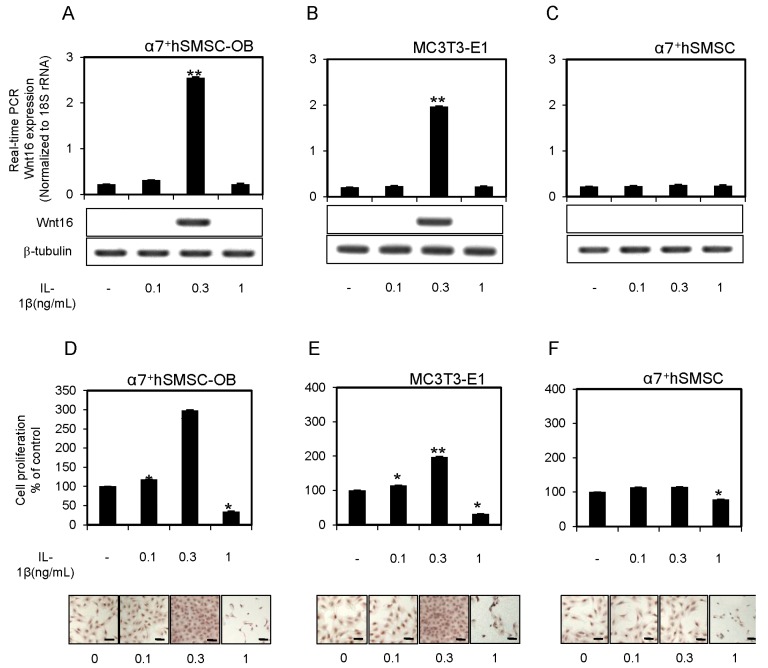
IL-1β-induced expression of Wnt16 mRNA and protein in α7^+^hSMSC-OB cells, MC3T3-E1 cells, and α7^+^hSMSCs. (**A**–**C**) Cells were treated with IL-1β (0, 0.1, 0.3 and 1 ng/mL), followed by real-time qPCR analysis of Wnt16 mRNA expression relative to the control (18S rRNA). Data are the mean ± SD of four independent experiments. ** *p* < 0.01. Western blot analysis of Wnt16 and β-tubulin protein levels was performed following stimulation with IL-1β (**lower** panels). Blots shown are representative of three independent experiments; (**D**–**F**) Effect of exogenous IL-1β on cell proliferation in α7^+^hSMSC-OB cells, MC3T3-E1 cells, and α7^+^hSMSCs. The effect of exogenous IL-1β on cell proliferation as evaluated using the BrdU-cell proliferation ELISA and BrdU immunohistochemistry kit that stains the nuclei of proliferating cells (dark brown; **bottom** panels). Scale bars = 100 μm. The cells were incubated in the absence or presence of IL-1β (0, 0.1, 0.3 and 1 ng/mL) for 24 h. Data are presented as the mean ± SD of four independent experiments. * *p* < 0.05, ** *p* < 0.01 *vs.* control.

α7^+^hSMSC-OB cells, MC3T3-E1 cells, and α7^+^hSMSCs were cultured in the presence of four concentrations of IL-1β (0, 0.1, 0.3 and 1 ng/mL). Induction of Wnt16 mRNA and protein was assessed by real-time quantitative polymerase chain reaction (qPCR) (Figure 1A–C) and Western blot analysis (lower panels), respectively. Both the mRNA and protein levels of Wnt16 were increased by IL-1β at 0.3 ng/mL, but not 0.1 or 1 ng/mL (Figure 1A,B). However, a different profile was observed in α7^+^hSMSCs (Figure 1C). Bone-associated cells also express other Wnt proteins such as Wnt3a, Wnt5a, Wnt5b, Wnt6, Wnt10a and Wnt11 [18,19,20,21,22]. To assess whether induction of Wnt16 by IL-1β is a specific response in α7^+^hSMSC-OB cells, we evaluated the expression of these other Wnt mRNAs and proteins following treatment with the same concentrations of exogenous IL-1β (0, 0.1, 0.3 and 1 ng/mL). However, there were no significant increases in these other Wnt mRNAs in response to IL-1β (Supplementary Material, Figure S1).

### 2.2. IL-1β Affects the Proliferation of α7^+^hSMSC-OB Cells

The lower concentrations of exogenous IL-1β (0.1 and 0.3 ng/mL) significantly increased cell proliferation (*p* < 0.05, *p* < 0.01; Figure 1D,E) in both α7^+^hSMSC-OB and MC3T3-E1 cells as determined by enzyme-linked immunosorbent assay (ELISA) and microscopic analyses. However, a different profile was observed in α7^+^hSMSCs (Figure 1F). In contrast, 1 ng/mL IL-1β drastically reduced cell proliferation (*p* < 0.05) and significantly increased apoptosis in both cell types (data not shown). These results demonstrate an inverse correlation between cell proliferation and apoptosis in α7^+^hSMSC-OB and MC3T3-E1 cells treated with IL-1β.

### 2.3. Expression of Lrp5 and Fzd2 mRNAs in α7^+^hSMSC-OB Cells

**Figure 2:**
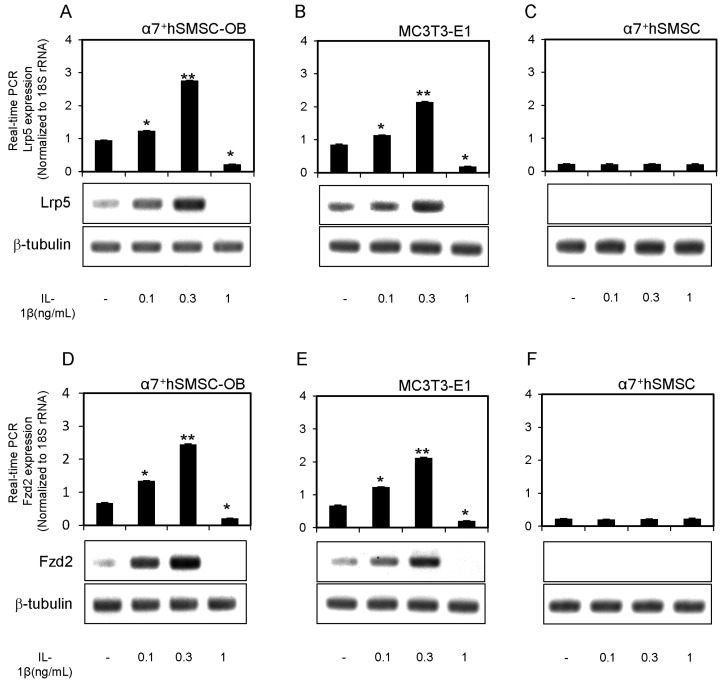
Expression of Lrp5 and Fzd2 mRNAs and proteins in α7^+^hSMSC-OB cells, MC3T3-E1 cells, and α7^+^hSMSCs. (**A**–**F**) Cells were stimulated with IL-1β (0, 0.1, 0.3 and 1 ng/mL) prior to real-time qPCR analysis of Lrp5 and Fzd2 expression relative to the control (18S rRNA). Data are the mean ± SD of four independent experiments. * *p* < 0.05, ** *p* < 0.01. Lower panels show Western blot analysis of Lrp5, Fzd2, and β-tubulin protein levels following stimulation with IL-1β. Blots are representative of three independent experiments.

We examined whether a Wnt receptor, Lrp5 and Fzd2, was present in α7^+^hSMSC-OB cells, MC3T3-E1 cells, and α7^+^hSMSCs, and whether its expression was influenced by IL-1β. Lrp5 mRNA and protein were constitutively expressed in α7^+^hSMSC-OB cells, and their levels were elevated by IL-1β treatment (0.1 and 0.3 ng/mL; Figure 2A). However, several other Wnt5 receptors are known, including Lrp6, Ror1, Ror2, Ryk, and Fzd9 [23,24,25]. We therefore measured the mRNA and protein expression of these Wnt5 receptors in our osteoblast-like cells. There was no evidence of Lrp6, Ror1, Ror2, Ryk, or Fzd9 expression in unstimulated or IL-1β-stimulated cells (Supplementary Material, Figure S2). Conversely Fzd2 was constitutively expressed in unstimulated cells, and its mRNA and protein levels were both increased by low-to-moderate concentrations of IL-1β (0.1 and 0.3 ng/mL; Figure 2D). IL-1β had no effects on Lrp5/Fzd2 expression in α7^+^hSMSCs (Figure 2C,F). Similar results were obtained from MC3T3-E1 cells (Figure 2B,E).

### 2.4. Effect of Exogenous Wnt16 on MMP-13 Expression and Cell Proliferation

**Figure 3:**
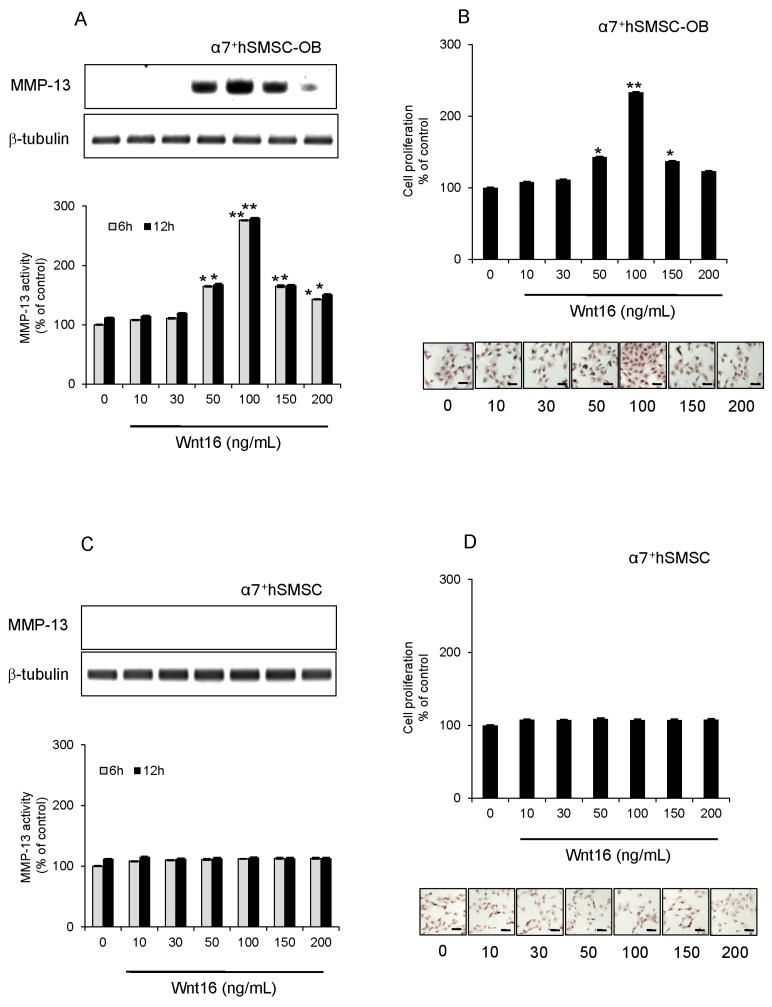
Effect of exogenous Wnt16 on MMP-13 expression and activity, and the proliferation of α7^+^hSMSC-OB cells. (**A**,**B**) Cells were treated for 6 h (grey bars) and 12 h (black bars) with 0, 10, 30, 50, 100, 150 and 200 ng/mL exogenous Wnt16 prior to analysis. Upper panels show Western blots of MMP-13 protein expression compared with β-tubulin levels. Blots are representative of three independent experiments; lower panels show MMP-13 activity. Data are the mean ± SD of four independent experiments * *p* < 0.05 and ** *p* < 0.01; (**C**,**D**) Effect of exogenous Wnt16 on cell proliferation. Cells were treated with Wnt16 (0, 10, 30, 50, 100, 150, and 200 ng/mL) for 24 h. Their proliferation status was then evaluated using the BrdU-cell proliferation ELISA (graphs) and BrdU immunohistochemistry kit (images; nuclei are stained brown in proliferating cells). Scale bars = 100 μm. ELISA data are the mean ± SD of four independent experiments. * *p* < 0.05, ** *p* < 0.01.

**Figure 4:**
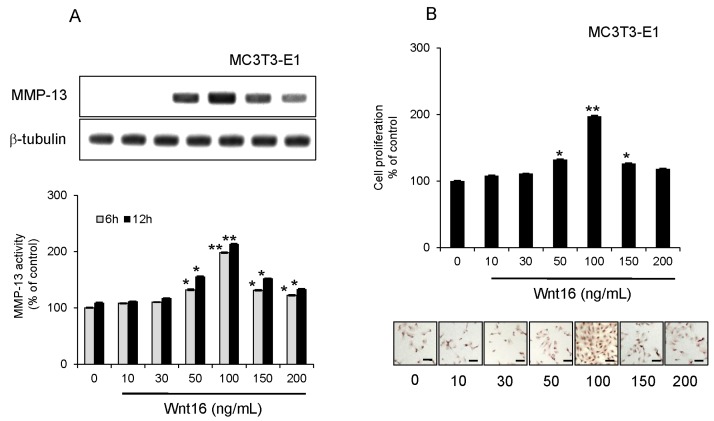
Effect of exogenous Wnt16 on MMP-13 expression and activity, and the proliferation of MC3T3-E1 cells. (**A**) Cells were treated for 6 h (grey bars) and 12 h (black bars) with 0, 10, 30, 50, 100, 150 and 200 ng/mL exogenous Wnt16 prior to analysis. Upper panels show Western blots of MMP-13 protein expression compared with β-tubulin levels. Blots are representative of three independent experiments. Lower panels show MMP-13 activity. Data are the mean ± SD of four independent experiments. * *p* < 0.05, ** *p* < 0.01; (**B**) Effect of exogenous Wnt16 on cell proliferation. Cells were treated with Wnt16 (0, 10, 30, 50, 100, 150 and 200 ng/mL) for 24 h. Their proliferation status was then evaluated using the BrdU-cell proliferation ELISA (graphs) and BrdU immunohistochemistry kit (images; nuclei are stained brown in proliferating cells). Scale bars = 100 μm. ELISA data are the mean ± SD of four independent experiments. * *p* < 0.05, ** *p* < 0.01.

In subsequent experiments, we used α7^+^hSMSC-OB cells with MC3T3-E1 cells as an authentic standard osteoblast-like cell type. We tested whether exogenous Wnt16 enhanced MMP-13 expression in osteoblast-like cells. MMP-13 protein expression and activity, as well as cell proliferation were all slightly increased by 50 ng/mL Wnt16 and dramatically increased by 100 ng/mL Wnt16 (*p* < 0.05; Figure 3A,C). However, the effect of 150 ng/mL Wnt16 was equivalent to that of 50 ng/mL Wnt16, indicating a significant decrease in efficiency at this higher dose (Figure 3A,C). Similar results were obtained from MC3T3-E1 cells (Figure 4A,B).

### 2.5. Effect of Anti-Wnt16 Small Interfering RNA (siRNA) on IL-1β-Induced Cell Proliferation

**Figure 5:**
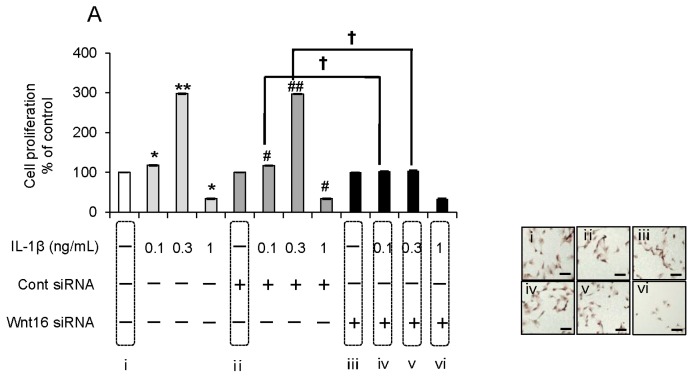

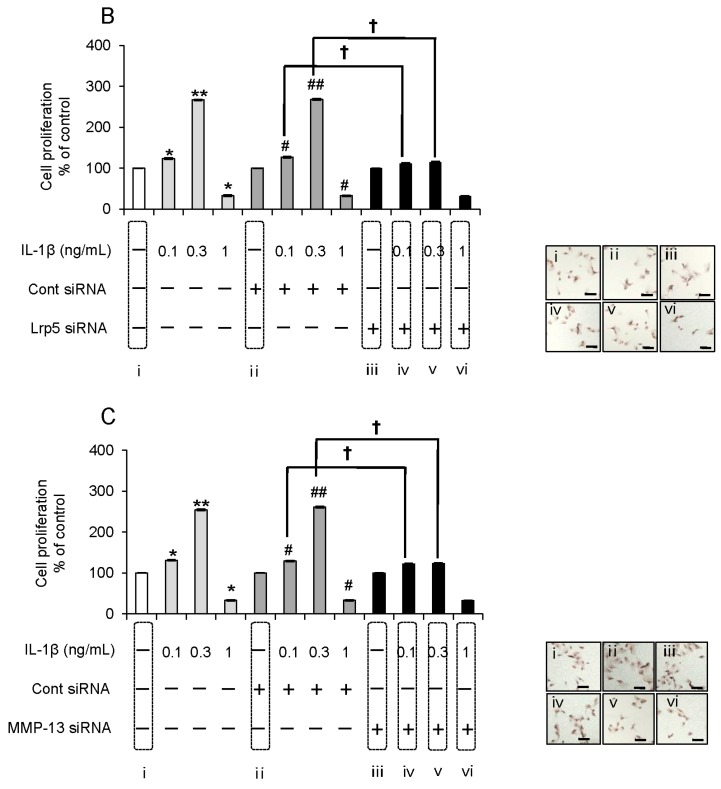
Effect of Wnt16, Lrp5, and MMP-13 siRNAs on IL-1β-induced cell proliferation. (**A**–**C**) α7^+^hSMSC-OB cells were transfected for 24 h with Wnt16, Lrp5, and MMP-13 siRNAs, stimulated with IL-1β (0, 0.1 0.3, and 1 ng/mL), and then analyzed by the BrdU-cell proliferation ELISA (graphs) and BrdU immunohistochemistry kit (images, **right** row). * *p* < 0.01 *vs.* control; ** *p* < 0.01 *vs.* control; ^#^ *p* < 0.01 *vs.* control siRNA; ^##^ *p* < 0.01 *vs.* control siRNA; ^†^ *p* < 0.01. The BrdU immunohistochemistry kit stains the nuclei of proliferating cells (dark brown). Images are representative of at least three independent experiments. Scale bars = 100 μm.

Under identical culture conditions, we tested the effect of anti-Wnt16 siRNA on IL-1β-induced changes in cell proliferation. Wnt16 silencing considerably decreased the number of proliferating α7^+^hSMSC-OB cells following IL-1β stimulation compared with untransfected and control siRNA-transfected cells. Similar results were obtained after siRNA-mediated knockdown of Lrp5 and MMP-13 (*p* < 0.01; Figure 5A–C). The reduction in the proliferative potential was estimated to be ~95%. These results were confirmed by microscopic analysis of cell proliferation (images in Figure 5A–C).

### 2.6. Evaluation of the Expression Order during IL-1β-Induced Cell Proliferation by siRNA-Mediated Silencing

**Figure 6:**
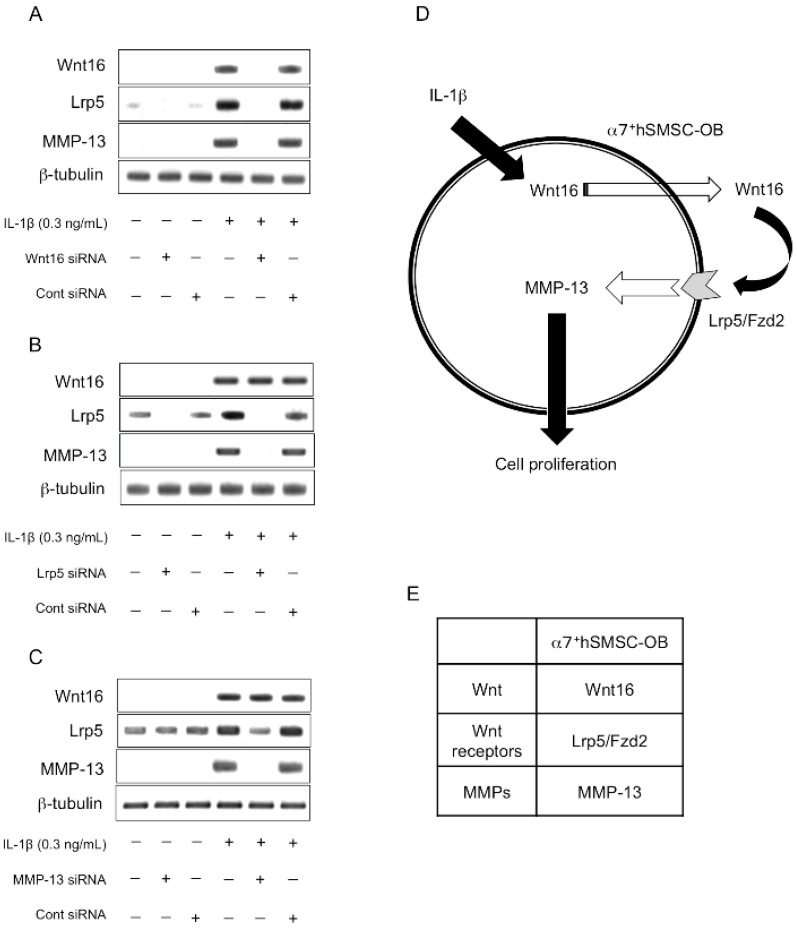
Determination of the signaling sequence using specific siRNAs. Cells were incubated for 24 h in serum-free medium containing IL-1β (0.3 ng/mL) and (**A**) Wnt16; (**B**) Lrp5; or (**C**) MMP-13 siRNAs. Protein expression of Wnt16, Lrp5, and MMP-13 was determined by Western blotting and compared with the expression of a housekeeping gene (β-tubulin). Images are representative of at least three independent experiments; (**D**) Schematic illustrating the putative signaling pathway by which IL-1β stimulates MMP-13 activity to induce the proliferation of α7^+^hSMSC-OB cells; (**E**) the different signaling cascades in IL-1β-induced proliferation of α7^+^hSMSC-OB cells.

Using several specific siRNAs, we examined the sequential order in which Wnt16, Lrp5, and MMP-13 are expressed in osteoblast-like cells by Western blot analysis (Figure 6A–C). IL-1β-induced expression of Wnt16 was only inhibited by anti-Wnt16 siRNA (Figure 6A), whereas Lrp5 expression was inhibited by both anti-Wnt16 and anti-Lrp5 siRNAs (Figure 6A,B). Furthermore, IL-1β-induced MMP-13 expression was inhibited by anti-Wnt16, anti-Lrp5, and anti-MMP-13 siRNAs (Figure 6A–C). Therefore, this signaling cascade appears to be IL-1β→Wnt16→Lrp5/Fzd2→MMP-13, and is intimately involved in the proliferation of α7^+^hSMSC-OB cells (Figure 6D).

## 3. Discussion

To date, functional data on the role(s) of Wnt16 signaling in human osteoblasts remain scarce. In the present study IL-1β was found to stimulate the Wnt16 signaling cascade, resulting in activation of MMP-13 and enhancement of osteoblast-like cell proliferation. This observation might represent a novel physiological function of Wnt16, and may be physiologically relevant to counteract the effects of inflammation *in vivo*.

Our data highlight three main points. First, this is the first study to use a specific siRNA targeting Wnt16 to elucidate the mechanism of IL-1β-induced proliferation in human skeletal muscle stem cell-derived, osteoblast-like cells. The lack of such a study until now is primarily because of the difficulty in generating sufficient numbers of purified human osteoblast-like cells. No study has investigated the effect of MMP-13 silencing on the proliferation of these cell types, except for our previous work [6]. The development of our basic knowledge with regard to human stem cell-derived osteoblastic cells is nevertheless highly important. Second, while Wnt5a and Wnt5b act as odontoblast growth factors [16], we demonstrated that Wnt16 is a growth factor for human osteoblastic cells. High concentrations of Wnt16 (>150 ng/mL) induced no cell proliferation, whereas lower concentrations (50–100 ng/mL) upregulated MMP-13 in these cells and increased cell proliferation. Because Wnt16 is a secreted protein, and its receptor (Lrp5/Fzd2) is present in these cells, it is possible that the addition of Wnt16 drove the proliferation of the osteoblastic cells, which might have led to wound healing *in vivo*. Therefore, targeting Wnt16 signaling in osteoblasts represents a promising approach to enhance the natural repair processes of bone fragility fractures and bone-destructive diseases such as RA, osteoporosis, and periodontitis. Finally, our most striking finding is that IL-1β induces an increase in Wnt16 signaling through Lrp5/Fzd2 as its receptor, leading to induction of MMP-13 expression and promotion of cell proliferation. *In vitro*, this activity is associated with an anti-inflammatory effect in osteoblast-like cells. Because this signaling cascade appears to be IL-1β→Wnt16→Lrp5/Fzd2→MMP-13, Wnt16 as a counter partner plays an important role in IL-1β-induced cell proliferation at a relatively early step in the signaling cascade. As shown in Figure 5, the combined weak inflammatory state induced by IL-1β and anti-Wnt16 siRNA resulted in potent suppression of cell proliferation, suggesting that Wnt16 expression might be a key determinant of cell survival. It remains to be established how IL-1β-induced MMP-13 activity regulates the proliferative effects observed in this study.

As a differentiation factor, endogenous Wnt signaling has functions in osteoblastogenesis and bone formation [26]. The importance of Wnt/β-catenin signaling in osteogenesis has been highlighted by the fact that (i) loss-of-function mutations in Lrp5, a co-receptor for Wnt/β-catenin signaling, cause osteoporosis and (ii) sclerostin, an osteocyte-derived inhibitor of Wnt/β-catenin signaling, suppresses bone formation [27]. A deficiency of Wnt10b, which is expressed in bone marrow, leads to reductions in trabecular bone mass, bone mineral density, and the serum osteocalcin level [28].

Wnt16 has been reported to induce the canonical Wnt pathway in osteoblasts [29]. Although we did not confirm that IL-1β induces the canonical Wnt pathway in human osteoblasts, we observed significant increases in Wnt16 mRNA expression, but not expression of Wnt3a, Win5a, Wnt5b, Wnt6, Wnt10a, or Wnt11 mRNAs in cells treated with IL-1β (data not shown). Furthermore, whereas IL-1β up-regulates the expression of MMP-1, -3, -9, and -13 via Wnt5a in rabbit TMJ condylar chondrocytes [23], we confirmed that IL-1β-mediated activation of Wnt16 in osteoblast-like cells led to induction of MMP-13 only (data not shown). Based on an analysis of Wnt16-deficient mice, it was concluded that Wnt16 mostly affects osteoclastogenesis directly and in an osteoprotegerin-dependent manner [29]. Because we have no definitive evidence, it remains to be elucidated.

Similarly, although numerous Wnt16 receptors exist (Lrp5, Lrp6, Ror1, Ror2, Ryk, Fzd2, and Fzd9) [30,31,32,33], we found that only Lrp5 and Fzd2 were induced by IL-1β in osteoblast-like cells (Figure 2) excepted for other Wnt16 receptors (data not shown). Because Lrp5 and Fzd2 are known to form a complex [34,35], this complex may act as the Wnt16 receptor in osteoblast-like cells. We previously reported that Lrp5/Fzd9 and Wnt5 are induced by IL-1β in odontoblast-like cells derived from mouse embryonic stem cells [16]; however, the different signaling cascade remains to be elucidated.

The effective dose range of IL-1β is very narrow (0.1 and 0.3 ng/mL). As described in our previous study [6], we concluded that, at lower IL-1β concentrations (*i.e.*, during the early stages of inflammation *in vivo*), MMP-13 contributes to tissue wound healing during osteoblast inflammation. It is likely that the switch from pro-proliferative to pro-apoptotic activity occurs as an instantaneous “step” rather than a gently “phased” shift. However, the physiological significance of this type of switch and the mechanism underlying it are unclear, and the reason why only low IL-1β levels can stimulate MMP-13 production in these cells remains to be elucidated.

## 4. Materials and Methods

### 4.1. Materials

Human and mouse recombinant IL-β were obtained from PeproTech (Rocky Hill, NJ, USA). Human and mouse recombinant Wnt16 were obtained from R & D Systems, Inc. (Minneapolis, MN, USA). Human and mouse ligands were used for human and MC3T3-E1 cells, respectively.

### 4.2. Cell Culture

α7^+^hSMSCs were a kind gift from Dr. Randall H. Kramer (University of California, San Francisco (UCSF), CA, USA) and maintained as described previously [17] with minor modifications. In brief, the cells were cultured in Ham’s F-10 medium containing 20% fetal bovine serum (FBS), 50 U/mL penicillin, 50 μg/mL streptomycin, 1 μg/mL insulin, 2.5 μg/mL fungizone, 0.5 mg/mL gentamicin, and 2 mM l-glutamine (all purchased from Invitrogen-Gibco, Carlsbad, CA, USA). This study was approved by the UCSF Committee on Human Research and the Aichi Gakuin University Ethics Committee (Approval No. 82, Approval Date: 21 September 2015). We had previously isolated human fetal and satellite muscle stem cells by taking advantage of the high expression of α7 integrin as a marker for the myogenic lineage [17]. These α7 integrin-positive fetal cells are capable of fusion and differentiate into myotubes with high efficiency. Therefore, fetal human cells can be easily purified and expanded *in vitro* to obtain large numbers of differentiation-competent myoblasts that might be suitable for differentiation into other cell types such as osteogenic cells. In our previous study, we found that bone morphogenetic protein-2 increases α2 integrin expression in differentiated α7^+^hSMSC-OB cells [17], which is concomitant with a loss of α7 integrin expression. Therefore, using flow cytometry, we evaluated the ratios of α2 integrin-positive cells as a percentage of the total population of differentiated cells to determine the purity of the differentiated cell population. Dead cells and debris were excluded by appropriate gating of forward and side scatter. Our α7^+^hSMSC-OB cell cultures were 98.33% ± 1.45% homogenous (% total, *n* = 3). We also detected the expression of osteoblastic markers osteopontin and osteocalcin in α7^+^hSMSC-OB cells, further indicating that the α7^+^hSMSCs had differentiated along the osteoblastic lineage (data not shown). We also confirmed specific osteogenic physiological changes in the cells (e.g., calcification and increased alkaline phosphatase activity), which continued for 21 days (data not shown). Mouse osteoblast-like MC3T3-E1 cells were purchased from the RIKEN BioResource Center Cell Bank (Ibaraki, Japan) and cultured as described previously [36,37]. These cells were used throughout the study as a positive control for the changes observed in human osteoblast-like cells.

### 4.3. Cell Proliferation Assay and Microscopic Analysis

Cell proliferation was evaluated using a 5-bromo-2′-deoxyuridine (BrdU)-cell proliferation ELISA (Roche Applied Science, Mannheim, Germany) as described previously [36,37]. The cells were seeded into 96-well culture plates at a density of 1 × 10^5^ cells/cm^2^. Cell proliferation was also evaluated visually with a BZ-9000 microscope (Keyence, Osaka, Japan) and/or IN Cell Analyzer 2000 (GE Healthcare UK Ltd., Buckinghamshire, UK) using a BrdU immunohistochemistry kit (Abcam, Cambridge, UK) according to the manufacturer’s instructions.

### 4.4. Real-Time qPCR Analysis

Real-time qPCR analyses were performed in triplicate in 96-well optical microtiter plates with ~25 ng RNA, 0.25 µL RT Mix (Qiagen Quantitect RT Mix, Valencia, CA, USA), 1.25 μL of 20× Primer/Probe Mix, and 12.5 μL Master Mix (Qiagen Quantitect RT-PCR kit) in a 25 μL reaction volume. The following primer/probe sets were used: human Wnt16, Mm00437347_m1; mouse Wnt16, Mm00437347_m1; human Wnt5a, Mm00437347_m1; mouse Wnt5a, Mm00437347_m1; human Wnt5b, Mm00437347_m1; mouse Wnt5b, Mm01183986_m1; human Lrp5, Mm01227476_m1; mouse Lrp5, Mm01227476_m1; human Fzd2, Mm02524776_s1; mouse Fzd2, Mm02524776_s1; human Fzd9, Mm01206511_s1; mouse Fzd9, Mm01206511_s1; human Lrp6, Hs00233945_m1; mouse Lrp6, Mm00999795_m1; human Ror1, Hs00938677_m1; mouse Ror1, Mm00443462_m1; human Ror2, Hs00896176_m1; mouse Ror2, Mm00443470_m1; human Ryk Hs00243196_m1; mouse Ryk, Mm01238551_m1 (Assays-On-Demand™, Applied Biosystems, Foster City, CA, USA). PCR samples were subjected to the following thermal cycling conditions: an initial holding stage of 30 min at 50 °C (for reverse transcription), 15 min at 95 °C (to activate HotStarTaq polymerase enzyme), and then 40 cycles of 15 s at 94 °C and 60 s at 60 °C. mRNA expression was quantified as relative to a standard curve. glyceraldehyde-3-phosphate dehydrogenase (GAPDH) and 18S amplicon rRNA were employed as housekeeping genes. For each experimental sample, the amounts of target and endogenous reference were determined from the appropriate standard curve. The amount of target was then divided by the amount of endogenous reference to obtain a normalized target value. *C*_t_ (threshold cycle) values for target and housekeeping genes were extrapolated from the standard curve to produce an arbitrary value of expression, the ratio of which (target/housekeeping gene) within a given tissue sample was plotted as the relative mRNA expression level.

### 4.5. Western Blot Analysis

Wnt16, Wnt5a, Wnt5b, Lrp5, Fzd2, Fzd9, Lrp6, Ror1, Ror2, Ryk and MMP-13 protein levels in cell lysates were determined by Western blot analyses. Cells were cultured for 6 h with or without IL-1β, lysed, and then protein lysates were separated on sodium dodecyl sulfate polyacrylamide gels (12%). Western blot analyses were then performed using anti-Wnt16, -Wnt5a, -Wnt5b, -Lrp5, -Fzd2, -Fzd9, -Lrp6, -Ror1, -Ror2, -Ryk, -MMP-13, and -β-tubulin polyclonal antibodies (sc-271897, sc-365370, sc-109464, sc-21390, sc-68328, sc-33509, sc-12363, sc-25317, sc-130867, sc-374174, sc-83080 and sc-9935, respectively; Santa Cruz Biotechnology Inc., Santa Cruz, CA, USA). The anti-MMP-13 antibody showed no significant cross-reactivity with other MMPs (data not shown). Visualization and quantification of blotted protein bands were performed with Multi Gauge-Ver3.X software (Fujifilm, Tokyo, Japan).

### 4.6. Measurement of MMP-13 Activity

The protocol to measure MMP-13 activity has been described previously [38,39] using a commercially available MMP-13 activity assay kit (SensoLyte^®^ Plus 520 MMP-13 assay kit AnaSpec, San Jose, CA, USA). MMP-13 was immunoprecipitated from the culture medium by incubation with an anti-MMP-13 goat antibody (sc-6839, Santa Cruz Biotechnology Inc.) and protein A/G-agarose for 6 h at 4 °C. After centrifugation, the agarose pellets were suspended in MMP-13 assay buffer from the kit (containing the MMP-13 substrates), and MMP-13 activity was determined according to the manufacturer’s instructions. 

### 4.7. Silencing of Wnt16, Lrp5, and MMP-13 Genes by siRNA Transfection

Wnt16, Lrp5, and MMP-13 siRNAs for gene silencing were acquired commercially (sc-41128, sc-43900 and sc-41559, respectively; Santa Cruz Biotechnology Inc.) and transfected into cultured cells using an siRNA reagent system (Santa Cruz Biotechnology Inc.) according to the manufacturer’s protocol. In brief, 2 × 10^5^ cells were seeded in 2 mL complete medium (Ham’s F-10 medium containing 20% FBS) in a 6-well plate. After 36 h, the cells were transfected with 66 pmol of the respective siRNA using the siRNA reagent system. We confirmed 96% inhibition achieved according to the manufacturer’s protocol. An anti-GAPDH siRNA and control siRNA (Thermo Scientific, Lafayette, CO, USA) without known homogeny for any vertebrate sequence were used as positive and negative controls, respectively.

### 4.8. Statistical, Analysis

Data are shown as the mean ± standard deviation (SD) of four to six independent experiments. Statistical significance was assessed using the Mann-Whitney *U*-test. A value of *p* < 0.05 was considered as statistically significant.

## 5. Conclusions

In summary, we have demonstrated that up-regulation of MMP-13 expression via the Wnt16 signaling pathway has an important role in the proliferation of human stem cell-derived, osteoblast-like cells. These results provide new insights into the role of Wnt16 in osteoblasts, and may have relevance to our understanding of, and ability to improve, wound healing following bone fragility fractures and bone-destructive diseases such as RA, osteoporosis, and periodontitis.

## Supplementary Materials

Supplementary materials can be found at http://www.mdpi.com/1422-0067/17/2/221/s1.
