# Supplementary material for: Wnt16 Signaling Is Required for IL-1β-Induced Matrix Metalloproteinase-13-Regulated Proliferation of Human Stem Cell-Derived Osteoblastic Cells
Source: Int J Mol Sci. 2016 Feb 6;17(2):221. doi: 10.3390/ijms17020221 (PMC4783953; doi:10.3390/ijms17020221)
Supplement: Supplementary file 1 [file ijms-17-00221-s001.pdf]

Supplementary Materials: Wnt16 Signaling Is Required for IL-1β-Induced Matrix Metalloproteinase-13-Regulated Proliferation of Human Stem Cell-Derived Osteoblastic Cells

Nobuaki Ozeki, Makio Mogi, Naoko Hase, Taiki Hiyama, Hideyuki Yamaguchi, Rie Kawai, Ayami Kondo and Kazuhiko Nakata

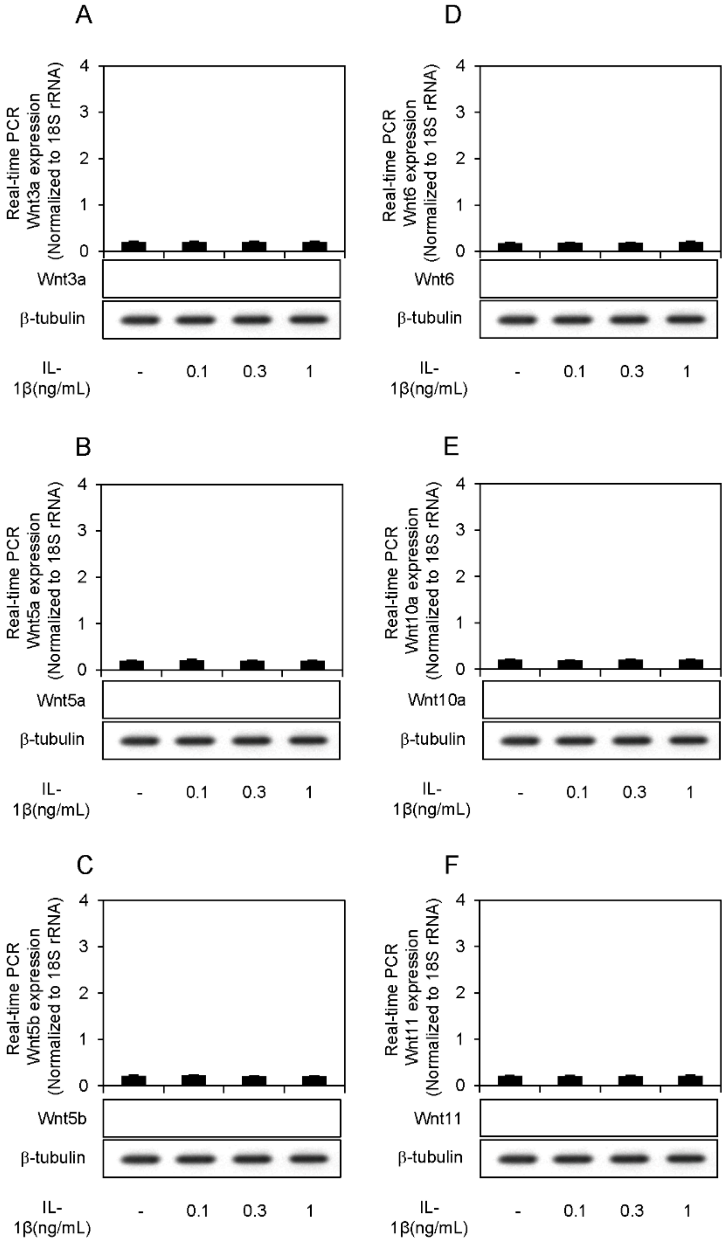

Figure S1. IL-1β-induced expression of Wnt3a, Wnt5a, Wnt5b, Wnt6, Wnt10a, and Wnt11 mRNAs and proteins in $\alpha7^{+}$hSMSC-OB cells. (**A**–**F**) Cells were treated with IL-1β (0, 0.1, 0.3 and 1 ng/mL), followed by real-time qPCR analysis of each Wnt mRNA relative to the control (18S rRNA). Data are the mean ± SD of four independent experiments. Western blot analysis of each Wnt protein and β-tubulin was performed following stimulation with IL-1β (**lower** panels). Blots shown are representative of three independent experiments.

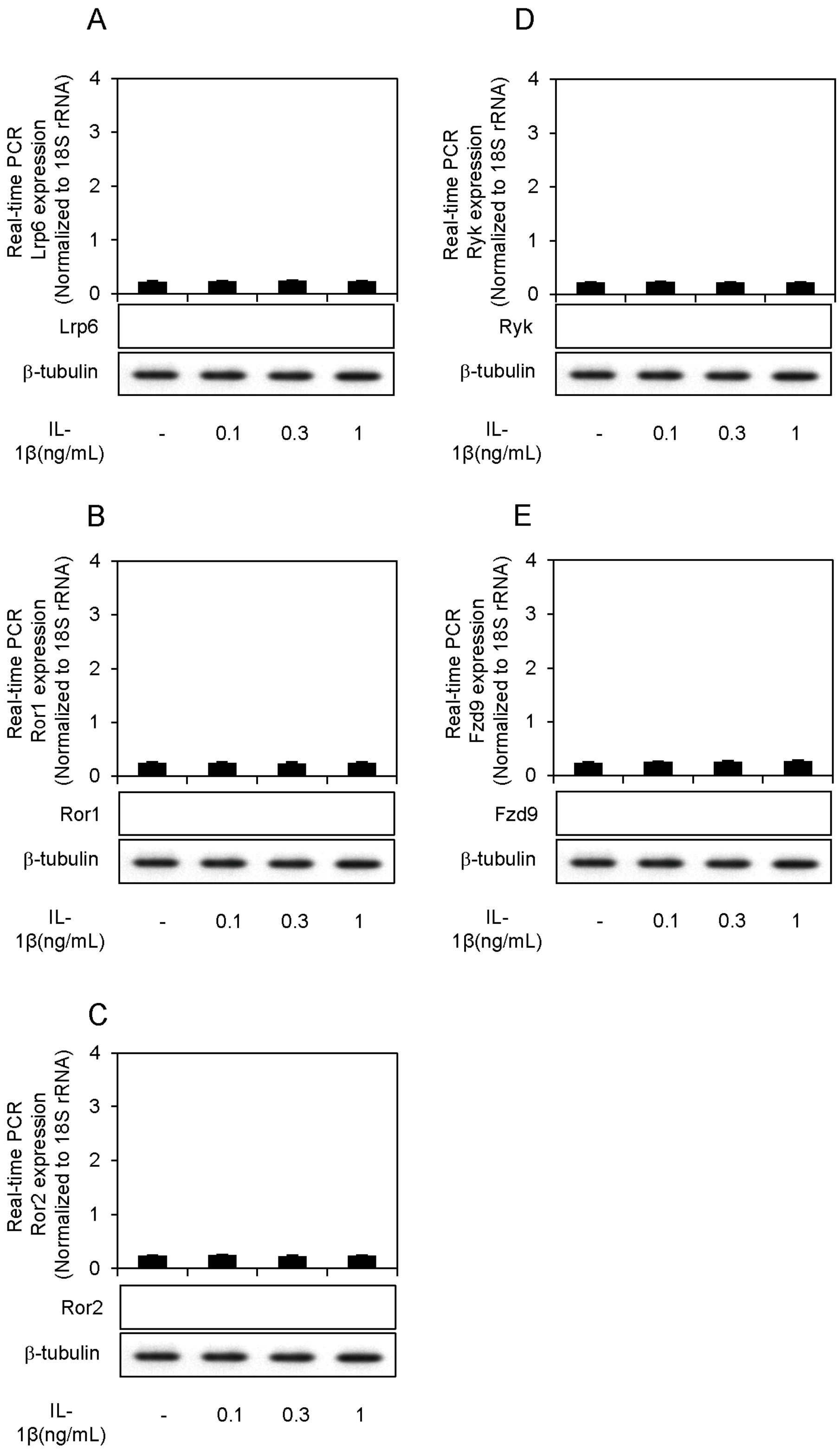

Figure S2. IL-1β-induced expression of Lrp6, Ror1, Ror2, Ryk, and Fzd9 mRNAs and proteins in $\alpha7^{+}$hSMSC-OB cells (**A**–**E**) Cells were treated with IL-1β (0, 0.1, 0.3 and 1 ng/mL), followed by real-time qPCR analysis of each mRNA relative to the control (18S rRNA). Data are the mean ± SD of four independent experiments. Western blot analysis of Lrp6, Ror1, Ror2, Ryk and Fzd9 and β-tubulin protein levels was performed following stimulation with IL-1β (**lower** panels). Blots shown are representative of three independent experiments.
